# Anti-GBM pulmonary-renal syndrome: when the laboratory makes the difference

**DOI:** 10.1515/almed-2025-0099

**Published:** 2026-01-12

**Authors:** Carmen Frías Ruiz, María D. Martín Martínez

**Affiliations:** Servicio de Análisis Clínicos, Hospital Universitario Nuestra Señora de Candelaria, Santa Cruz de Tenerife, Spain

**Keywords:** anti-glomerular basement membrane (anti-GBM), anti-GBM disease, goodpasture syndrome

## Abstract

**Objectives:**

Pulmonary-renal syndrome (PRS) is a serious condition characterized by concurrent alveolar hemorrhage and rapidly progressive glomerulonephritis. One of the potential causes of PRS is the presence of anti-glomerular basement membrane (anti-GBM) antibodies, which may induce Goodpasture syndrome, a rare, albeit life-threatening, condition associated with a high mortality.

**Case presentation:**

We report the case of a 31 year-old male patient with Huntington’s disease who presented with dyspnea, a dry cough, and asthenia. Urgent laboratory tests demonstrated severe anemia, acute kidney failure, and additional laboratory abnormalities consistent with vasculitis. Chest CT scan revealed diffuse alveolar hemorrhage. The autoimmune panel was anti-GBM positive, thereby leading to diagnosis of Goodpasture syndrome.

**Conclusions:**

Goodpasture syndrome is a form of pulmonary-renal syndrome that requires prompt diagnosis and intensive immunosuppressive therapy. Patients may follow a severe course and progress into end-stage renal disease. Early detection of anti-GBM antibodies is essential for diagnosis. The clinical laboratory plays a major role in supporting therapeutic decision-making and treatment response monitoring.

## Introduction

Pulmonary-renal syndrome is a severe condition defined as the combination of rapidly progressive glomerulonephritis and pulmonary hemorrhage, potentially inducing acute kidney failure and severe respiratory compromise. Leading causes include anti-neutrophil cytoplasmic antibody (ANCA) associated small-vessel vasculitis and anti-glomerular basement membrane (anti-GBM) disease, also known as Goodpasture syndrome [1]. ANCA-mediated vasculitis accounts for 60 % of all pulmonary-renal syndrome cases. This form of vasculitis is mediated by neutrophils activated in response to cytoplasmic proteins. In contrast, anti-GBM vasculitis, which is immunocomplex-mediated, accounts for around 15 % of all cases [2], 3].

Anti-GBM disease is a rare albeit potentially fatal autoimmune disease characterized by the presence of autoantibodies directed against the non-collagenous domain of the α3 chain of type IV collagen present in the glomerular and alveolar basement membrane. These autoantibodies activate the complement system, thereby triggering an inflammatory reaction that promotes neutrophil infiltration and the formation of cellular crescents in glomeruli. When not promptly treated, these events may cause irreversible kidney damage and alveolar hemorrhage. Although kidney dysfunction is the most serious manifestation of Goodpasture syndrome, it is the co-occurrence of respiratory and renal symptoms what defines this condition, having an incidence of 0.5 to 1 cases per one million population [4].

Disease progression may be fatal and cause rapid deterioration of kidney function, which may eventually require renal replacement therapy. Patients with Goodpasture syndrome and ANCA (around 20–35 %) may exhibit other clinical manifestations and require a specific therapeutic approach [4], 5].

## Case presentation

A 31 year-old male patient, former smoker, diagnosed with Huntington’s disease presented to the Emergency Department with a one-week history of oral aphthae, asthenia, cough without hemoptysis, and dyspnea on minimal exertion that hindered daily life activities. On physical examination, the most remarkable findings included BP of 140/70 mmHG, 93 % oxygen saturation, and fissures along the lateral borders of the tongue, whereas no edemas or signs of deep vein thrombosis were noted.

Urgent laboratory tests demonstrated: hemoglobin 5.6 g/dL (13,0–16,5); urea 203 mg/dL (74–100); creatinine 14.07 mg/dL (0.73–1.18); glomerular filtrate 4 mL/min/1.73 m^2^ (>90); Na 121 mmol/L (135–150); LDH 427 U/L (125–220); PCR 12.98 mg/dL (<0.5); procalcitonin 1.91 ng/mL (<0.5). In view of these findings, the patient was admitted to the Internal Medicine Ward (IMW) with a diagnosis of suspected vasculitis.

A chest CT scan demonstrated signs suggestive of extensive bilateral diffuse alveolar hemorrhage. Laboratory and imaging findings raised suspicion of pulmonary-renal syndrome secondary to vasculitis.

Autoimmunity panel testing was negative for antinuclear antibodies (ANA) and ANCA antibodies, while positive for anti-GBM antibodies at a titer>680 U/mL (<7 negative; 7–10 indeterminate; >10 positive) by fluorescent polarization immunoassay. These results were consistent with a diagnosis of Goodpasture syndrome, which was subsequently confirmed by histopathological analysis. The histoptahology report described extracapillary prolypherative glomerulonephritis with linear IgG deposits along the glomerular basement membrane in over 80 % of glomeruli added to intense IgG, Kappa and Lambda linear deposits by direct immunofluorescence.

While in the Internal Medicine and Nephrology ward, the patient received phasmapheresis therapy until the patient achieved anti-GBM-negative status, plus glucocorticoids and cyclophosphamide. Therapy was successful and respiratory symptoms subsided. However, persistent oligoanuric kidney failure made dialysis necessary throughout the entire hospital stay. Six months after starting cyclophosphamide therapy, the patient continues undergoing dialysis, with a glomerular filtration rate of 15 mL/min; creatinine 4.31 mg/dL. Renal biopsy demonstrated fibrotic crescents in over 85 % of glomeruli, which led to patient’s inclusion in the kidney transplant waiting list.

## Discussion

Goodpasture syndrome is a rare, life-threatening, form of pulmonary-renal syndrome. Rapid progression to multisystem failure and the need for an aggressive immunosuppressive therapy underline the relevance of timely diagnosis and the need for a multidisciplinary approach [2].

Patients often need intensive care due to severe organ failure. Despite appropriate management and therapeutic advances, prognosis continues to be guarded, with significant mortality rates and a high risk for progression to end-stage renal disease [3], 6].

Differential diagnosis should include other causes of pulmonary-renal syndrome, including granulomatosis with polyangiitis and microscopic polyangiitis. In this context, early determination of anti-GBM antibodies is essential for establishing a final diagnosis and an appropriate treatment. ANCA-positive patients may present different clinical manifestations and require a specific therapeutic approach. In our case, ANCA was ruled out thorough autoimmunity testing [7]

Although final diagnosis is confirmed by renal biopsy, the clinical laboratory plays a pivotal role in the initial diagnostic phase, enabling the early initiation of immunosuppressive therapy and the adoption of kidney protective measures that are essential for long term survival. Moreover, laboratory testing remains crucial during follow-up, allowing monitoring of disease until the patient achieves antibody-negative status [8]. This case underlines the relevance of integrating clinical, serological and histopathological findings to optimize therapeutic outcomes.

## Highlights

– Co-occurrence of pulmonary hemorrhage and acute kidney failure should raise suspicion of pulmonary-renal syndrome, since it may progress rapidly and require urgent treatment.
– Early detection of anti-GBM antibodies is key to establishing diagnosis of Goodpasture syndrome, especially in ANCA-negative cases.
– The role of the clinical laboratory is crucial, not only for initial diagnosis, but also for monitoring treatment response through autoantibody quantification.
– This case highlights the relevance of adopting a multidisciplinary approach by integrating clinical, laboratory, imaging and histopathological data to ensure an optimal management of rare autoimmune diseases.
